# Vitamin D levels in patients of acute leukemia before and after remission-induction therapy

**DOI:** 10.12669/pjms.291.2764

**Published:** 2013

**Authors:** Arshi Naz, Rizwan N. Qureshi, Tahir S. Shamsi, Tabassum Mahboob

**Affiliations:** 1 Arshi Naz, M.Sc, M.Phil, Department of Hematology, National Institute of Blood Disease and Bone Marrow Transplantation, Karachi, Pakistan.; 2 Rizwan N. Qureshi, MBBS, FCPS, Department of Internal Medicine, National Institute of Blood Disease and Bone Marrow Transplantation, Karachi, Pakistan.; 3 Tahir S. Shamsi, MBBS, FRCPath, Department of Clinical Hematology and Oncology, National Institute of Blood Disease and Bone Marrow Transplantation, Karachi, Pakistan.; 4 Tabassum Mahboob, PhD, Department of Biochemistry, University of Karachi, Karachi, Pakistan

**Keywords:** AL: Acute Leukemia, ALL: Acute Lymphoblastic Leukemia, AML: Acute Myeloid Leukemia, Vitamin D

## Abstract

***Objectives:*** To determine the levels of 25-hydroxyvitamin [25(OH)D3] in patients with acute leukemia and the effect of remission-induction chemotherapy.

***Methodology: ***This study was case control, all newly diagnosed patients of acute leukemia between the age of one to sixty years and residents of Pakistan were enrolled and evaluated. Those who were unwilling or unable to provide written informed consent were excluded. All selected patients (n=86) were grouped in to acute myeloid leukemia (AML) and acute lymphoblastic leukemia (ALL). AML was further categorized as A1 before remission-induction (n=17) and B1 after remission induction (n=13), ALL was further categorized as A2 before remission-induction (n=31) and B2 after remission induction (n=25). The 25-hydroxyvitamin [25(OH)D3] levels were measured in the sera of all patients (before and after remission-induction) by one step delayed chemiluminescent micro particle immunoassay (CMIA).We compared 25(OH)D3 levels in all patients before and after the remission-induction chemotherapy.

***Results: ***A total of 86 patients were analyzed, in which 60 patients were male. Mean age was 24.39 years (range, 1 to 60 years); the mean levels of 25(OH)D in group A1 (n=17) was 17.70±3.2 ng/ml, in group B1 (n=13) 14.06±2.4 ng/ml, 19.07±7.08 ng/ml in group A2 (n=31), while 10.59±3.9 ng/ml found in group B2 (n=25).

***Conclusion: ***25(OH)D3 insufficiency was evident subnormal in majority of patients with acute leukemia and 25(OH)D3 were further reduced after remission-induction as compared to untreated group, difference was statistically significant when compared with each group.

## Introduction

 Vitamin D deficiency is common in Pakistan. Multiple studies in different parts of the country demonstrated that 70% to 97% asymptomatic healthy individuals were identified vitamin D deficient.^1^^-^^3^ Vitamin D is obtained either from skin exposure to ultraviolet B radiation in the form of sunlight, or through dietary sources including supplementation. Serum levels of 25-hydroxyvitamin D [25(OH)D3] reflect whole body vitamin D stores, and are used to assess individual adequacy or insufficiency. 25(OH)D3 is converted to 1,25-dihydroxyvitamin D [1,25(OH)2D], considered the physiologically active form of vitamin D, via the action of 1-α-hydroxylase. While much of this conversion occurs in the kidney, multiple other tissues (including lymphoma tumor cells) also have 1- α -hydroxylase activity, and can thus regulate 1,25(OH)2D levels at the local tissue level. Once formed, 1,25(OH)2D exerts its effects through binding to the vitamin D nuclear transcription factor receptor, where it may regulate the expression of nearly 200 genes.^4^

 In addition to its role in calcium and bone homeostasis, vitamin D potentially regulates many other cellular functions, which include differentiation, apoptosis, angiogenesis, and immunoregulation. Prevalence of vitamin D level monitoring has significantly increased as the awareness of its potential importance to health has increased.^5^ At present, the strongest data for an inverse association between circulating vitamin D levels and malignancy exists for studies that have examined patients with colorectal^6^^,^^7^ and breast^8^^,^^9^ cancer.

 However, serum level of vitamin D have been less evaluated in acute leukemias including acute myeloid leukemia (AML) and acute lymphoblastic leukemia (ALL). Hun Ju Lee et al studied vitamin D levels in 97 cases of AML and found 63 (65%) patients had levels <32ng/ml. 34 (35%) patients had levels considered insufficient (20-31.9ng/ml), 29 (30%) patients were deficient (<20ng/ml) and 34 (35%) patients had normal vitamin D levels (32-100 ng/mL).^10^ Pardanani et al reported vitamin D insufficiency in myeloproliferative neoplasms, and noted severe 25(OH)D deficiency in essential thrombocythemia (12%) and primary myelofibrosis (9%), compared with polycythemia (3%) and myelodysplastic syndrome (1%) (P = 0.05).^11^ Another study reported that in acute leukemias, vitamin D deficiency appeared very common and had inverse relation to therapy: lower levels being related to poorer prognosis.^12^

 Simmons JH et al investigated 78 survivors of acute lymphoblastic leukemia, (52 chemotherapy-treated and 26 hematopoeitic stem cell-treated). This study reported 53% of pediatric ALL survivors were 25-hydroxyvitamin D insufficient (15-29 ng/dl), and 12% were deficient (<15 ng/dl), and also concluded that there was no significant difference in serum 25-hydroxyvitamin D status between chemotherapy-treated and HSCT-treated ALL survivors. Even this study has found that the serum levels of 25-hydroxyvitamin D were similar to general pediatric population.^13^ The purpose of this study was to determine the frequency of levels of 25-hydroxyvitamin D in acute leukemia in Pakistani population.

## Methodology

***Setting:*** This study was carried out in the Department of Biochemistry, University of Karachi, Pakistan and Department of Pathology and Haem Oncology, National Institute of Blood Disease and Bone Marrow Transplantation, Karachi, Pakistan during May 2011 to March 2012. Study protocol was approved by institutional review board.

***Study Population: ***A written informed consent was obtained from all participants. Since May 2011, we offered enrollment to consecutive, newly diagnosed patients with acute leukemia (within 120 days) who were evaluated, were aged 1-60 years. Exclusion criteria included unwillingness or inability to provide written informed consent. All pathology was reviewed by a leukemia hematopathologist to verify the diagnosis and to classify each case according to the WHO classification.^14^

***Sample Size:*** All selected patients (n=86) were grouped in to acute myeloid leukemia and acute lymphoblastic leukemia (ALL). AML was further categorized as A1: before remission-induction (n=17) and B1: after remission induction (n=13), ALL also was further categorized as A2: before remission-induction (n=31) and B2: after remission induction (n=25).

 Baseline clinical, laboratory, and treatment data were abstracted from medical records using a standard protocol. Participants provided a peripheral blood sample for serum banking. Timing of the sample collection with respect to treatment (i.e., before and after remission-induction) was recorded. All patients were systematically observed during each cycle of chemotherapy. Disease progression and deaths were verified through medical record review. 

***Collection & Storage of Blood Specimen:*** 5cc venous blood was drawn from each patient on presentation of disease and after receiving remission-induction. Transferred each sample into tube which had no additive. Serum was separated by centrifugation at 3000 rpm for 10 minutes at room temperature within one hour of blood collection. Samples were stored in aliquots at -80C^0^ until assayed. All samples were run in batches of 30.

***Vitamin D Measurements:*** We defined vitamin D insufficiency as a serum 25(OH)D3 level lower than 29 ng/mL(62.5 nmol/L) and deficiency of vitamin D was defined levels less than 10 ng/mL. Although consensus guidelines for the diagnosis of vitamin D insufficiency have not been established, this is an accepted level for the establishment of hypovitaminosis D. Quantitative 25-Hydroxy Vitamin D was determined by using one step delayed chemiluminescent micro particle immunoassay (CMIA) [Abbot Diagnostics (Germany)] and levels were expressed in nanogram/milliliter (ng /mL).

***Data Analysis:*** Simple descriptive analysis was applied for demographic variables. An independent T-test was used to test significant difference between the cases of both acute leukemias. One way analysis of variance (ANOVA) was applied to test the mean differences between the sub-types of AML and ALL patients. Statistical analysis was carried out using Statistical Package for the Social Sciences (SPSS) version 17.0.

## Results

 Eighty-six patients with acute leukemia were analyzed in which 60 were male; mean age was 24.39 years (range 1-60 years). In all, 30 (34.88%) patients were diagnosed as acute myeloid leukemia and 56 (65.11%) were with the diagnosis of acute lymphoblastic leukemia.

**Fig.1:**
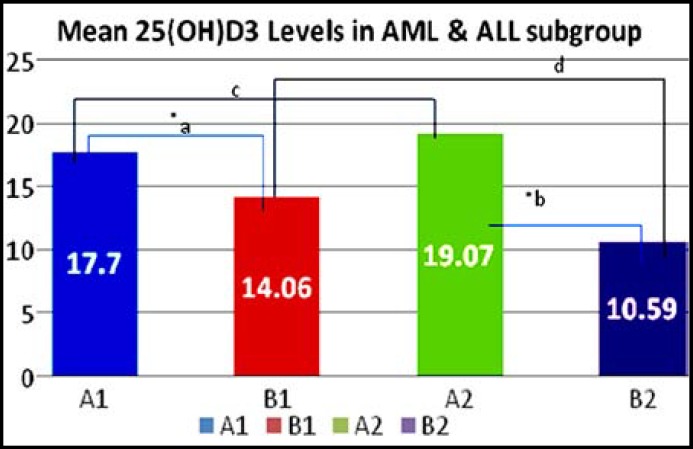
Comparison of vitamin D [25(OH)D] levels between groups of acute leukemia.

Fig.1: Comparison between 25(OH)D levels in group A1, B1, A2 & B2. Numerical values of vitamin D3 level in sera of patients with acute leukemia are shown as Mean+S.E. alphabetical notations indicate comparison between two groups./ *show level of significance P<0.05. A1 Before remission-induction acute lymphoblastic leukemia, B2: After remission-induction acute lymphoblastic leukemia. aA1 vs. B2, bA2 vs.B2, cA1 vs. A2, dB1 vs. B2. *Shows level of significance P<0.05.

 The mean levels of 25(OH)D in group A1 (n=17) had 17.70±3.2 ng/ml, in group B1 (n=13) 14.06±2.4 ng/ml, 19.07±7.08 ng/ml in group A2 (n=31), while 10.59±3.9 ng/ml found in group B2 (n=25) (Fig.1). The 25(OH)D levels in all patients were reported below 29 ng/ml, which was defined as insufficient levels except in A1 (n=2) and A2 (n=1) had levels between 30-100 ng/ml. Significant reduction noted in patients received remission-induction therapy in both acute leukemias but marked difference was found in ALL group.

 In “A1” group (before remission-induction AML) 5 (5.8%) patients had 25(OH)D levels <10 ng/ml (deficient); mean was 13.7 (±5.6) ng/ml, 10(11.6%) had levels between 10-29 ng/ml (insufficient); mean was 16.2 (±6.1) and 2(2.3%) had levels between 30-100 ng/ml; mean was 26.3 (±21.7) ng/ml respectively. While in “B1” group (after remission-induction AML) 3(3.5%) patients had 25(OH)D levels <10 ng/ml (deficient); mean was 16.2 (±9.0) ng/ml, 10(11.6%) patients had 10-29 ng/ml (insufficient); mean was 14.3 (±5.7) and none had normal levels (30-100ng/ml).

 In “A2” group (before remission-induction ALL) 8 (9.3%) patients had 25(OH)D levels <10 ng/ml (deficient); mean was 30.2 (±10.2) ng/ml, 21 (24.4) had levels between 10-29 ng/ml (insufficient); mean was 15.7 (±9.4) and 1 (1.2%) had levels between 30-100 ng/ml; mean was 19.4 (±3.0) ng/ml respectively. While in “B2” group (after remission-induction AML) 11 (12.8%) patients had 25(OH)D levels <10 ng/ml (deficient); mean was 14.4 (±5.7) ng/ml, 14 (16.3) patients had 10-29 ng/ml (insufficient); mean was 27.5 (±19.3) and none had normal levels (30-100ng/ml).

**Fig.2:**
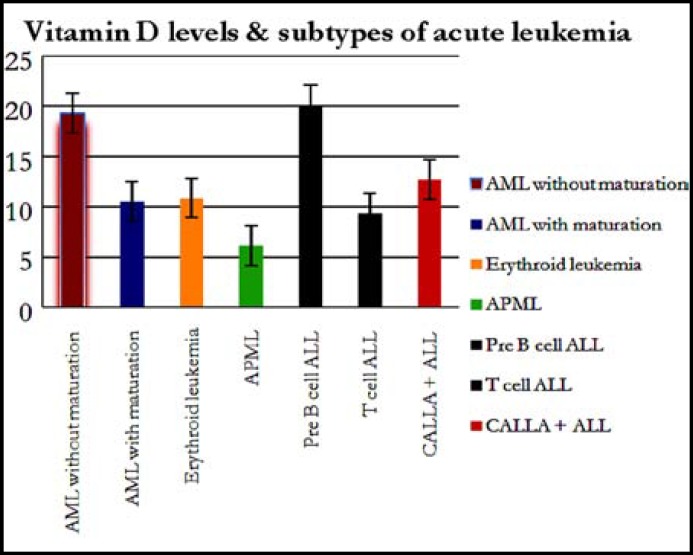
Vitamin D levels in different types of acute leukemia

Fig.2: Mean levels of vitamin D3 are expressed as mean + SE in subtypes of acute lymphoblastic leukemia (CALLA positive, T cell ALL, pre B cell ALL) and acute myeloid leukemia (acute promyelocytic leukemia, acute erythroid leukemia, myelomonocytic leukemia, AML with maturation & AML without maturation). One way ANOVA was applied for testing of significance level p<0.05.

 25(OH)D (Fig.2) levels were also determined in subtypes of acute leukemia (both in ALL & AML); AML without maturation (n=19) had 15.88±6.0 ng/ml, AML with maturation (n=3) had 8.4 ±3.27 ng/ml, myelomonocytic (n=1) had 11.7±0.99 ng/ml, erythroid leukemia had (n=4) 11.6±0.28 ng/ml, APML (n=3) had 10.3 ±3.3 ng/ml, pre-B cell leukemia (n= 23) had 16.16±16.15 ng/ml, T-cell leukemia (n=8) had 31.74±52.25 ng/ml, CALLA positive ALL (n=25) had 11.74±7.23 ng/ml of 25(OH)D levels (Fig.2). 

**Table-I:** Different levels of Vitamin D [25(OH)D] in different groups of acute leukemia.

*25(OH)D Levels (ng/ml)*	*AML*						*ALL*					
	**A1**			**B1**			**A2**			**B2**		
	n (%)	Mean 25(OH)D Levels (ng/ml)	p *	n (%)	Mean 25(OH)D Levels (ng/ml)	p *	n (%)	Mean 25(OH)D Levels (ng/ml)	p*	n (%)	Mean 25(OH)D Levels (ng/ml)	p*
<10	5 (5.8)	13.7 (±5.6)	0.21	3 (3.5)	16.2 (±9.0)	0.66	8 (9.3)	30.2 (±10.2)	0.67	11 (12.8)	14.4 (±5.7)	0.28
10-29	10 (11.6)	16.2 (±6.1)		10 (11.6)	14.3 (±5.7)		21 (24.4)	15.7 (±9.4)		14 (16.3)	27.5 (±19.3)	
30-100	2 (2.3)	26.3 (±21.7)		-	-		1 (1.2)	19.4 (±3.0)		-		
>100	-	-		-	-		1 (1.2)	-		-		
Total	17			13			31			25		

Table shows the differences between groups of acute leukemia according to vitamin D [25(OH)D] levels, defined as <10 ng/ml: Deficient levels, 10-29: Insufficient levels, 30-100: Normal levels, >100: High normal levels. All quantitative variables are expressed as mean±SD. N shows number of participants. Vitamin D3 insufficiency is represented in par enthesis as percentage. Level of significance was *p*<0.05.

## Discussion

 In acute leukemia, the evidence based association between vitamin D and acute leukemia to date provides limited support. However there was an exception noted when Lee HJ et al.^10^ have recently reported 25(OH)D3 levels at the time of diagnosis of AML and their association with survival, found worse progression free survival in AML patients with subnormal 25(OH)D3. Simmons JH et al.^13^ found low vitamin D levels in ALL but concluded no significant difference in treated and untreated patients. There are several reports (Lee HJ^10^ 2010; Drake MT et al^15^ 2010; Shanafelt TD et al^16^ 2011) indicating that low levels of vitamin D have been shown to be associated with worse clinical outcome; however, there are no prospective studies evaluating whether supplementation would improve outcome.

 In our study, over 90% of patients with acute leukemia (including AML, ALL) had insufficient 25(OH)D levels. 25(OH)D insufficiency was more pronounced in ALL patients (p=0.56) as compared to AML. 25(OH)D levels significantly reduced after remission-induction chemotherapy in ALL population as in AML (p <0.05). This finding was contradicting the results of Lee HJ^10^ study. Patients with AML with maturation found to had lowest levels in all leukemia while T-cell leukemia had the highest levels of 25(OH)D3. These associations remained after adjustment for clinical factors, timing of blood draw and season of diagnosis. These estimates were not statistically significant, most likely due to either the small number of patients with these types of acute leukemia included in the study, and had relatively short follow-up time.

 Our study had some strengths and limitations. The strengths include prospective study design of consecutively enrolled patients with newly diagnosed acute leukemia (AML, ALL) availability of baseline clinical and treatment data and complete follow-up of patients of 28 days. In addition, we used chemiluminescent microparticle immunoassay (CMIA) methods for the measurement of serum 25(OH)D and 1,25(OH)2D levels, and a technique which is considered to be the most reliable and accurate for 25 (OH) D determinations. The major limitation of the study is the use of an observational study design, which is susceptible to confounding, although we were able to adjust for key clinical prognostic factors. On these results we can suggest future interventional studies as the patients outcome including performance status, will be analyzed by administering exogenous vitamin D supplements.

## Conclusions

 25(OH)D3 insufficiency was evident subnormal in majority of patients with acute leukemia and 25(OH)D3 were further reduced after remission-induction as compared to untreated group, difference was statistically significant when compared with each group. 
